# 1-Trichloromethyl-1,2,3,4-tetrahydro-beta-carboline (TaClo) Induces the Apoptosis of Dopaminergic Neurons via Oxidative Stress and Neuroinflammation

**DOI:** 10.1155/2019/1292891

**Published:** 2019-03-07

**Authors:** Yihang Yang, Bo Pang, Zihao Liu, Jie Li, Zhen Zhang, Rui Zhang, Xianzeng Hou, Hua Guo, Lingyi Chi, Qi Pang, Tao Xin

**Affiliations:** ^1^Department of Neurosurgery, Shandong Provincial Hospital Affiliated to Shandong University, Jinan, Shandong 250021, China; ^2^Department of Neurosurgery, Qilu Hospital of Shandong University, Jinan, Shandong 250012, China; ^3^Medical College of Nanchang University, Nanchang, Jiangxi 330000, China; ^4^Department of Neurosurgery, Qianfoshan Hospital affiliated to Shandong University, Shandong 250014, China; ^5^Department of Neurosurgery, Qilu Hospital of Shandong University and Brain Science Research Institute of Shandong University, Jinan, 250012 Shandong, China; ^6^Department of Neurosurgery, Jiangxi Provincial People's Hospital Affiliated to Nanchang University, Nanchang 330006, Jiangxi, China

## Abstract

Several *in vitro* studies have revealed the neurotoxicity of 1-trichloromethyl-1,2,3,4-tetrahydro-beta-carboline (TaClo). However, the underlying mechanism has not been completely elucidated, particularly *in vivo*. This study was designed to study the neurotoxicity of TaClo *in vivo* by stereotactically injecting TaClo into the striatum of Wistar rats. After the TaClo injections, rats were subjected to an open field test, and their distance travelled and tracks showed decreasing trends over time. The results of liquid chromatography-mass spectrometry analysis showed that the motor dysfunction of the TaClo-treated rats was accompanied by reduced dopamine levels in the striatum. Based on the diffusion tensor imaging data, the apparent diffusion coefficient of the nigrostriatal pathway was significantly increased, and subsequent histological staining revealed the demyelination of nigrostriatal fibres after the TaClo treatment. TaClo induced a loss of tyrosine hydroxylase-positive cells in the substantia nigra compacta. Regarding the underlying mechanism, TaClo caused oxidative stress in the nigrostriatal system by increasing the production of reactive oxygen species and reducing the mitochondria membrane potential. Meanwhile, the elevated expression of Iba-1, TNF-*α*, IL-6, Cox-2, and iNOS indicated microglial activation and a strong innate immune response in the nigrostriatal system. In addition, activated caspase-3 levels were increased. Thus, both mitochondrial impairments and the innate immune response are involved in TaClo-induced neurotoxicity.

## 1. Introduction

Parkinson's disease (PD) is one of the most common neurodegenerative movement disorders and affects approximately 1-2% of elderly people. Both genetic and environmental factors are strongly correlated with the development of PD. However, to date, the exact aetiology and underlying molecular mechanisms of PD remain largely unclear [1]. 1-Methy-l-4-phenyl-1,2,3,6-tetrahydropyridine (MPTP) is known to cause PD. Many other structural analogues of MPTP have been discovered in the environment, including herbicides (e.g., paraquat), alkaloids (e.g., 1,2,3,4-tetrahydroisoquinolines), and *β*-carbolines [2]. Trichloroethylene (TCE) is widely used as a detergent, extractant, and solvent. The main routes of TCE exposure are industrial waste gas, contaminated groundwater, and volatile organic solvents. 1-Trichloromethyl-1,2,3,4-tetrahydro-beta-carboline (TaClo) is an *in vivo* metabolic product of TCE. TaClo belongs to the *β*-carboline family and has a structure similar to the synthetic neurotoxin 1-methyl-4-phenylpyridinium iodide (MPP^+^) [3]. Previous studies have reported TaClo concentrations ranging from less than 1 ng to 35 ng per millilitre in blood samples from patients who were orally treated with chloral hydrate for 3 days to 6 months [4, 5]. Moreover, TaClo is approximately 10 times more potent than MPTP [6], and it penetrates the blood-brain barrier more easily than MPTP [7]. However, to date, the mechanism by which TaClo induces PD remains unclear.

The nigrostriatal pathway (NP) is an anatomical circuit comprising dopaminergic neurons that project from the substantia nigra compacta (SNc) to the striatum forming part of the basal ganglia motor loop. Dopaminergic neurons in the SNc and their terminals in the striatum constitute the nigrostriatal dopaminergic system. This system is easily impaired because many extracellular materials can be taken up via plasma membrane dopamine transporters [8]. We hypothesized that TaClo causes PD symptoms by damaging the nigrostriatal system. Mitochondrial dysfunction, oxidative stress, and inflammation are involved in degenerative diseases [9]. As shown in the study by Prof. Bing G., TCE treatment impairs mitochondrial complex I activity [10]. Furthermore, by referring to the GEO database (GSE7621-GPL570), which provides analyses of the SNc in postmortem brain tissues from patients, the expression of the Cox-2, iNOS, GABPA, and Keap 1 genes is upregulated in patients with PD. We also hypothesized that TaClo induces a positive feedback loop between mitochondrial oxidative stress and the innate immune response in the central nervous system (CNS). Mitochondrial inhibitors, such as rotenone and MPTP, are known to increase reactive oxygen species (ROS) production by blocking the electron transport chain at a particular site [11], further causing abnormalities in neuroglia [12]. More importantly, substantially elevated ROS levels are implicated in the activation of the innate immune response [13]. Subsequently, activated microglia release ROS, further impairing the mitochondria. This type of positive feedback between mitochondrial dysfunction and the inflammatory cascade in the brain may cause secondary injury, such as demyelination, dopaminergic neuron apoptosis, and neurodegenerative diseases.

## 2. Materials and Methods

### 2.1. Experimental Animals

Fifty-five male Wistar rats aged between 8 and 10 weeks (weight 260-300 g, purchased from Vital River Laboratory Animal Technology Co. Ltd., Beijing, China) were included in this experimental study. All animals were housed under standard conditions with a 12 h day/night cycle and free access to food and water (room temperature 22°C, humidity 55%). The experimental animals were randomly divided into two groups: a control group (*n* = 25; 272.3 ± 9.6 g) and a TaClo group (*n* = 25; 276.9 ± 7.8 g). Rats in the TaClo group were stereotactically injected with TaClo (provided by the laboratory of Dr. Bing Guoying, USA) dissolved in polyethylene glycol (PEG) (P3015, Sigma-Aldrich, St Louis, MO, USA) into the right striatum. The control rats were injected with PEG. All procedures were performed in accordance with the guidelines and regulations of the Experimental Centre of Shandong Provincial Hospital and approved by the Ethics Committee of Shandong Provincial Hospital affiliated to Shandong University.

### 2.2. Stereotactic Injection

Rats (*n* = 25; 276.9 ± 7.8 g) were anaesthetized with an intraperitoneal injection of ketamine. After being anaesthetized, the animals were placed in a stereotaxic apparatus (ZH-B, Zhenghua Biological Instrument Equipment Co., Ltd, China). The stereotactic injections were performed with a 1 *μ*l microsyringe (Hamilton, Sweden). The following stereotactic coordinates were used for the unilateral intrastriatal injections relative to the bregma: site 1: anteroposterior (AP) 1.0 mm, mediolateral (ML) 2.0 mm, and dorsoventral (DV) 5.5 mm; site 2: AP 1.0 mm, ML 3.5 mm, and DV 6.0 mm; site 3: AP -0.5 mm, ML 2.5 mm and DV 5.0 mm; and Site 4: AP -0.5 mm, ML 4.0 mm and DV 6.5 mm. TaClo was dissolved in PEG at a concentration of 2.50 *μ*g/*μ*l. Considering that rats weighing 250 g to 350 g have similar brain volumes, each rat was injected with the same amount of TaClo corresponding to 1 *μ*l per injection site. The total dose was 10 *μ*g of TaClo per rat [14]. The injection rate was 0.25 *μ*l/min, and the syringe remained in the injection site for an additional 5 min before being retracted. After the injection, each rat was monitored until it recovered from the anaesthesia. The control animals (*n* = 25; 272.3 ± 9.6 g) were stereotactically injected with PEG. Rats in the sham group (*n* = 3; 269.5 ± 6.3 g) received only surgery; they did not receive any injections and served as a control to exclude possible effects of the surgical procedures on the results of the diffusion tensor imaging (DTI) test.

### 2.3. Open Field Test

Rats (*n* = 5) were recorded with an automatic video tracking system (ZH-ZFT, Zhenghua Biological Instrument Equipment Co. Ltd, China). Rats in both the TaClo group and the control group were placed in the testing box (40 × 40 × 35 cm) in a random order. Before the test, each rat was allowed to adapt to its surroundings for 10 min. Then, during the following 30 min, the movements of the rats, including the tracks and total distance travelled, were recorded by the video tracking system.

### 2.4. DTI

Rats (*n* = 3) were anaesthetized before the test. The test was performed by an imaging technician. Conventional MRI and DTI scans were conducted on a 3.0 T MRI scanner (Philips Achieva TX, Best, The Netherlands) with a wrist coil. Rats were placed in the scanner in the prone position. Conventional MRI scans, including T1-weighted and T2-weighted sagittal images, were completed with the SE sequence. DTI images were acquired using a spin-echo planar imaging sequence. After acquiring the images, the data were transferred to a Philips workstation to calculate the DTI data. For accurate positioning, the DTI images were mixed with sagittal T2-weighted images and the regions of interest (ROIs) in the striatum and the SNc were defined. The parameters, including the apparent diffusion coefficient (ADC) and fractional anisotropy (FA) values, were analysed. The averages of multiple measurements were considered the final results.

### 2.5. Luxol Fast Blue (LFB) Staining

Rats were euthanatized with carbon dioxide. The brains were removed, fixed with 4% paraformaldehyde for 48 h, and then embedded in paraffin. The sections were cut at a thickness of 10 *μ*m using a microtome and stained with LFB to reveal the demyelinated areas according to the manufacturer's protocol (American MasterTech, CA, USA).

### 2.6. Liquid Chromatography-Tandem Mass Chromatography (LC–MS/MS) Analysis

Rat striatal tissues weighing 50–60 mg were homogenized in a solution containing 200 *μ*l of ultrapure water (0.1% formic acid) and 800 *μ*l of ice-cold methanol/acetonitrile (1 : 1, *v*/*v*). The homogenate was vortex-mixed for 1 min and ultrasonically lysed on ice for 30 min. Then, the homogenate was centrifuged at 14,000 × *g* for 20 min at 4°C. The supernatant was transferred to a new tube and evaporated to dryness under a nitrogen stream. The dry residue was reconstituted in 100 *μ*l of the initial mobile phase (0.1% formic acidin water/acetonitrile, 1 : 1, *v*/*v*) and centrifuged at 14,000 × *g* for 15 min. Then, the supernatant was injected into the LC–MS system for analysis. The MS-mediated acquisition of dopamine (DA), *γ*-amino-butyric acid (GABA), epinephrine (Epi), glutamine, glutamate, 5-hydroxyindole-3-acetic acid (5-HIAA), and serotonin (5-HT) spectra was performed in the electrospray positive ionization multiple reaction monitoring (MRM) mode.

### 2.7. Immunofluorescence (IF) Staining

For the IF analysis, frozen sections were permeabilized with 0.2% Triton X-100 for 20 min and blocked with goat serum for 1 h. Then, the slides were incubated with a rabbit monoclonal anti-tyrosine hydroxylase (TH) primary antibody (1 : 200, ab75875, Abcam, USA) at 4°C overnight. The slides were washed with PBS 3 times for 5 min each and incubated with an Alexa 488-conjugated goat-anti-rabbit secondary antibody (1 : 200, ab150077, Abcam, USA). The excitation wavelength used for the assay is 490 nm, and the emission wavelength is 520 nm.

### 2.8. Nissl Staining

Paraffin sections were routinely dewaxed and incubated with toluidine blue in a 55°C incubator for 40 min. Sections were washed with distilled water 3 times for 5 min each. After differentiation with hydrochloric acid in alcohol, sections were dehydrated and sealed with neutral gum.

### 2.9. Flow Cytometry (FCM) Analysis

For staining, the tissue was minced and digested with trypsin for 15 min at 37°C. Then, the tissue homogenate was filtered through a 40 *μ*m filter and prepared using a fixation/permeabilization solution according to the manufacturer's instructions (BD Pharmingen, San Diego, CA). Cells were incubated with a primary rabbit-anti-rat TH (1 : 200, ab75875, Abcam) at 4°C for 40 min. After washes with PBS, cells were incubated with a goat-anti-rabbit-PE secondary antibody (1 : 100, ab7010, Abcam, USA). The cells were washed with PBS again. Then, cells were stained with a ROS dye (Invitrogen, Carlsbad, USA) at 37°C for 1 h according to the manufacturer's protocol. After washes with PBS, multiple colour fluorescence-activated cell sorting (FACS) analyses were performed by using a FACSCalibur cytometer (Beckman Coulter, KBB, CA, USA). The data were analysed using FlowJo software (TreeStar, San Carlos, CA). First, the TH-positive cells were sorted. The ROS mean fluorescence intensity in these TH-positive cells was measured and analysed.

### 2.10. JC-1 Staining

The brains were immediately embedded in O.C.T (Tissue-Tek, Sakura, USA). In addition, 10 *μ*m sections were cut using a freezing microtome. Changes in the mitochondrial membrane potential were measured by incubating the tissues with 5,5′6,6′-tetrachloro-1,1′3,3′-tetraethylbenzimidazole-carbocyanine iodide (JC-1, Beyotime Biotechnology, China) for 20 min at 37°C. Aggregates of the fluorescent JC-1 probe yield red fluorescence with an emission wavelength of 590 nm. In addition, the monomer form of the fluorescent JC-1 probe yields green fluorescence with an emission wavelength of 530 nm.

### 2.11. ATP Measurement

ATP levels were measured using a luciferin-luciferase-based ATP assay kit (Beyotime, China). The working solution was prepared according to the manufacturer's protocol. One hundred microliters of working solution was added to each well of a 96-well plate. The 96-well plate was incubated at room temperature for 5 min. Then, the ATP standards and samples were added. Luciferase activity was detected with a microplate reader (Synergy H1, BioTek, USA). The ATP level was calculated according to the standard curve, normalized to the protein contents in each sample and reported as nmol/mg of protein.

### 2.12. Immunohistochemistry (IHC)

Rats (*n* = 3) were euthanized and perfused with 150 ml of saline and 300 ml of 4% paraformaldehyde. Brain tissues were collected and fixed with 4% paraformaldehyde for 48 h. Using a rat brain mould, brain tissues were cut at the same coronal plane of the SNc. Then, 10 *μ*m-thick coronal slices were prepared. Antigens were retrieved in citric acid buffer (pH 6.0) using a high-pressure antigen retrieval method. Every sixth coronal section from the region was incubated with rabbit anti-Iba-1 (1 : 500, 019-19741, Wako, Japan) and rabbit monoclonal anti-*α*-synuclein primary antibodies (1 : 200, ab51253, Abcam, USA) at 4°C. Then, the immunoreactive cells were visualized using streptavidin-peroxidase-conjugated goat-anti-rabbit secondary antibody (SP kits, PV-9000, ZSGB-Bio Co. Ltd., Beijing, China). In addition, the mean integrated optical density (IOD) of the positive staining was measured using Image-Pro Plus software (Media Cybernetics, USA). Six equal areas from each section were randomly selected to measure the area of positive staining. Five sections from each sample were counted, and the average value was calculated.

### 2.13. Quantitative Real-Time PCR

Total RNA was isolated using TRIzol reagent (Invitrogen). Alterations in the mRNA levels of selected genes were measured using RT-PCR following the reverse transcription of 1 *μ*g of RNA from each sample using a PrimeScript RT reagent kit (Takara Bio Inc., Otsu, Japan). Quantitative RT-PCR was performed on a LightCycler instrument (Roche Diagnostics, Mannheim, Germany) with SYBR Premix Ex Taq technology (Takara Bio Inc., Otsu, Japan) according to the manufacturer's protocol. The relative mRNA levels were determined using the 2^-ΔCt^ method. The primer sequences used to determine the expression of specific genes are listed in Table 1.

### 2.14. Western Blot Analysis

An equal amount of proteins (40 *μ*g) was separated using SDS-polyacrylamide gel electrophoresis and transferred to polyvinylidene fluoride membranes. Membranes were blocked with 5% skim milk and incubated with primary antibodies against TH (monoclonal, 1 : 10000, ab75875, Abcam, USA), TNF-*α* (monoclonal 1 : 1000, 17590-1-AP, Proteintech, USA), IL-6 (monoclonal, 1 : 1000, sc-57315, Santa Cruz, USA), Cox-2 (monoclonal, 1 : 1000, 12282, CST, USA), iNOS (polyclonal, 1 : 500, 18985-1-AP, Proteintech, USA), and caspase-3 (polyclonal 1 : 1000, 19677-1-AP, Proteintech, USA) overnight at 4°C. Membranes were then incubated with the appropriate secondary antibodies (anti-rabbit IgG, ZB-2301, and anti-rat IgG, ZB-2305, ZSGB-BIO, China) at room temperature for one hour and washed with TBST three times. Membranes were visualized using a Digital Imaging System and analysed using AlphaView software.

### 2.15. Equipment and Settings

The western blotting results were obtained using a luminescent image analyser (Amersham Imager 600, Sweden). An optical microscope (Leica DM4000 B, Germany) was used to capture the images of IHC staining. A fluorescence microscope (Carl Zeiss A2, Germany) was used to capture images of the IF, ROS, and JC-1 staining. Images were processed using AlphaView SA, Image-Pro Plus, GraphPad Prism, and Photoshop without using touch-up tools, such as cloning and healing tools.

### 2.16. Statistical Analysis

We used SPSS version 19.0 to analyse the data in this study. The differences in the ADC and FA values among the three groups were analysed using one-way ANOVA followed by Fisher's LSD multiple comparison tests. Additionally, Student's *t* test was performed to compare the data between the TaClo and control groups. All tests were two-tailed. Statistical significance was defined as *p* < 0.05. The data are presented as means ± standard deviations (SD) of at least three independent experiments.

## 3. Results

### 3.1. Rats Exhibited Gradual Symptoms of Bradykinesia after TaClo Injection

**Figure 1:**
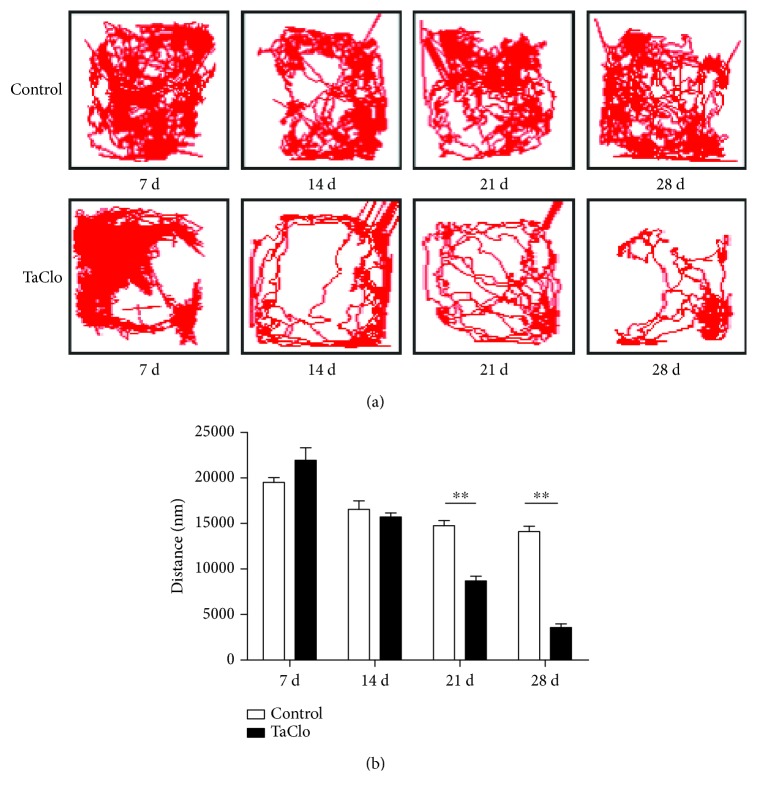
TaClo induced motor ability impairments. (a and b) The open field test revealed that symptoms of bradykinesia in the TaClo-injected rats on the 21st and 28th days after injection compared to the control rats. ^∗∗^ *p* < 0.01 compared to the control group.

In the TaClo group, the distance travelled in the open field test showed an evident decreasing trend over time. The ranges of the tracks travelled by the rats in the TaClo group narrowed towards a corner compared to those travelled by the rats in the control group (Figure 1(a)). In addition, the rats in the TaClo group travelled a significantly shorter distance than did the rats in the control group on the 21st and 28th days (Figure 1(b)). The motor activity decreased by approximately 78.1% (95% CI, 71.3% to 82.5%) on the 28th day after TaClo treatment. In contrast, rats in the control group did not show any significant difference in the distance travelled at different time points (*p* > 0.05). Based on the video recording, the frequency of spontaneous rotary motion increased on the 28th day after TaClo treatment (Supplementary File 1). According to the results of the behavioural test, the rat PD model was successfully established on the 28th day after TaClo treatment. Therefore, we chose this time point for further study.

### 3.2. TaClo Induced Demyelination in the Nigrostriatal System

**Figure 2:**
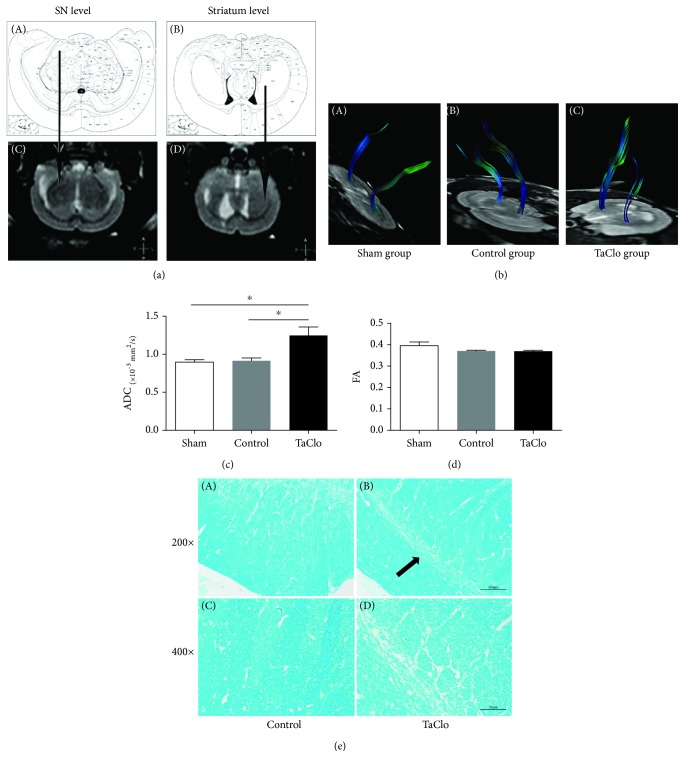
TaClo impaired the nigrostriatal system. (a) Comparison of stereotaxic coordinates in an atlas of the rat brain and the ROI assessed using DTI. Arrows pointing from A to C and from B to C show the SNc and striatum, respectively. (b) Images of the general outline of the nigrostriatal system indicate the obvious destruction of this region in the TaClo-injected rats compared to the other two groups. (c) Histogram showing a significant increase in the ADC value for the nigrostriatal system after TaClo injection. ^∗^ *p* < 0.05 compared to the control group. ^∗^ *p* < 0.05 compared to the sham group. (d) The FA value for the NP did not significantly change. (e) LFB staining showing demyelination in the SNc of the TaClo-treated rats (arrow).

On the 28th day, DTI was performed to examine the nigrostriatal system in the tested rats. In the TaClo group, images of the general outline of the NP showed obvious destruction in the right nigrostriatal cluster, which was not observed in the sham and control groups (Figures 2(a) and 2(b)). Furthermore, the ADC values for the NP were significantly increased in the TaClo-treated rats. No significant changes in the ADC values were detected in the sham and control groups. The FA values for the NP did not differ among the three groups (Figures 2(c) and 2(d)). Based on these DTI results, TaClo impaired the integrity of the nigrostriatal system. Furthermore, the LFB staining revealed demyelination of dopaminergic neurons in the TaClo group consistent with the DTI results (Figure 2(e)).

### 3.3. TaClo Decreased the Dopamine Level and Induced the Loss of Dopaminergic Neurons in the Nigrostriatal System

**Figure 3:**
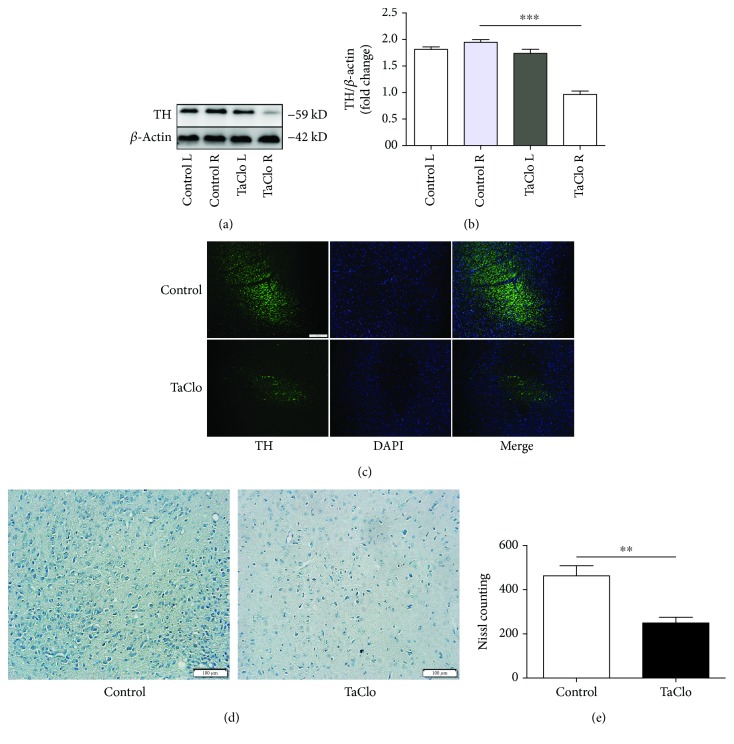
TaClo decreased dopamine levels and induced a loss of TH-positive cells in the nigrostriatal system. (a and b) Western blots showing the TH levels in the nigrostriatal system on the injected and untreated side in both control and TaClo-treated rats on the 28th day. ^∗∗^ *p* < 0.01 compared to the control group. (c) Images of IF staining showing TH expression in the SNc in the two groups. (d and e) Staining for Nissl bodies in rats from two two groups. ^∗∗^ *p* < 0.01 compared to the control group. Scale bar 100 *μ*m.

LC–MS/MS was performed to determine whether the motor dysfunction exhibited by the TaClo-treated rats was accompanied by a decreased striatal DA level. TaClo significantly decreased the DA level in the striatum. The levels of other types of neurotransmitters, including serotonin, glutamate, glutamine, 5-HIAA, and tyramine, did not differ between the two groups (Table 2). The western blot showed significantly decreased TH levels in the nigrostriatal system after TaClo injection (Figures 3(a) and 3(b)). Meanwhile, the IF images of the full SNc area revealed an apparent loss of TH-positive cells after TaClo treatment (Figure 3(c)). Nissl bodies in the nigrostriatal system were apparently decreased after the TaClo injection (Figures 3(d) and 3(e)). TaClo impaired the function of dopaminergic neurons in the nigrostriatal system *in vivo*.

### 3.4. TaClo Induced Mitochondrial Dysfunction by Causing Oxidative Stress

**Figure 4:**
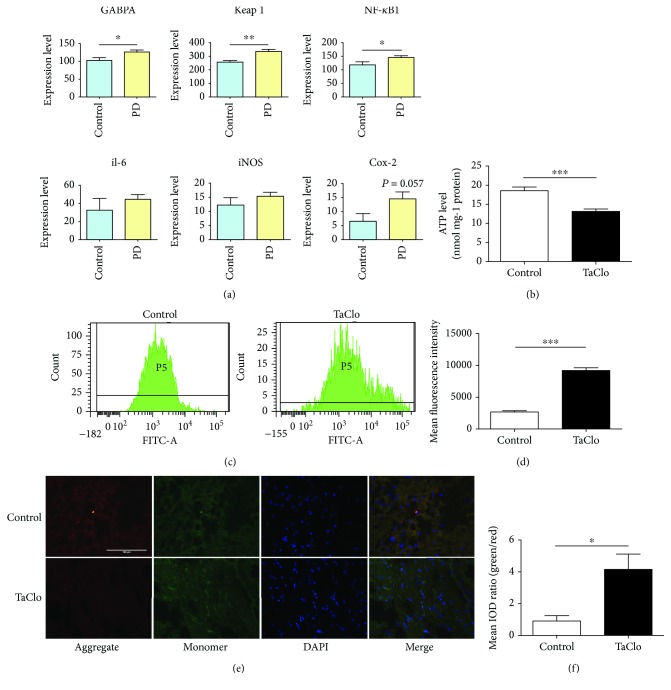
TaClo caused mitochondrial oxidative stress in the nigrostriatal system. (a) The expression of representative genes related to oxidative stress and inflammation from the GEO database (GSE7621-GPL570) was analysed. (b) ATP production was reduced after TaClo treatment. ^∗∗^ *p* < 0.01 compared to the control group. (c and d) FCM revealed an apparent increase in the MFI of ROS in the TH-positive cells from the TaClo-treated group compared to the control group. (e and f) Results of JC-1 staining in the two groups. The ratio of green and red fluorescence was increased after the TaClo treatment. ^∗^ *p* < 0.05 compared to the control group. Scale bar 100 *μ*m.

Subsequently, we investigated the mechanism by which TaClo impairs dopaminergic neurons *in vivo*. By referring to gene expression data obtained from patients with PD in the GEO database (GSE7621-GPL570), which included an analysis of the SNc from patients with PD, the expression of both oxidative stress- and inflammation-related genes was upregulated in the SN of patients with PD (Figure 4(a)). TaClo also impairs the function of mitochondrial complex I. Therefore, we postulated that the negative effect of TaClo on dopaminergic neurons was associated with oxidative stress. The ATP measurements showed decreased ATP levels after the TaClo treatment (Figure 4(b)). The results of FCM analysis revealed a significantly higher mean fluorescence intensity (MFI) of ROS in the TH-positive cells from the TaClo group than in the control group (Figures 4(c) and 4(d)). These results indicated that TaClo caused oxidative stress in the nigrostriatal system. TaClo impaired the normal function of the mitochondria in dopaminergic neurons. JC-1 staining revealed a decrease in the mitochondrial membrane potential, which is known to occur at the early stage of cell apoptosis (Figures 4(e) and 4(f)).

### 3.5. TaClo Induced the Innate Immune Response and Apoptosis in the Nigrostriatal System

**Figure 5:**
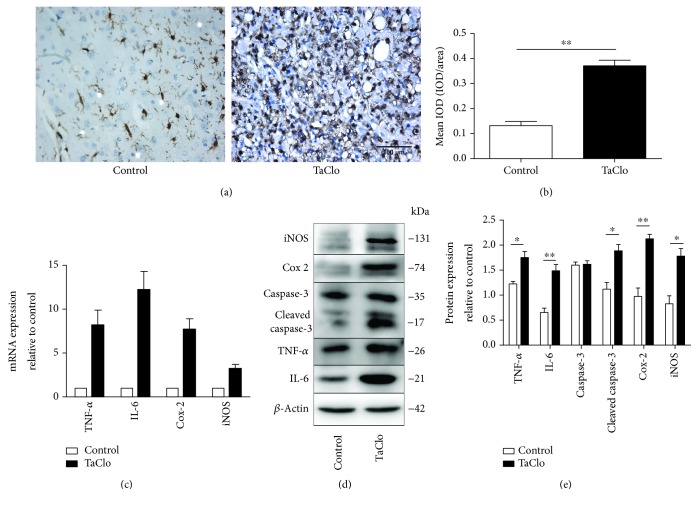
The TaClo injection triggered inflammation in the nigrostriatal system. (a) Images of IHC staining showing significantly increased Iba-1 expression in the nigrostriatal system after the TaClo injection (400x). ^∗∗^ *p* < 0.01 compared to the control group. (b) RT-PCR results increased levels of the TNF-*α*, IL-6, Cox-2, and iNOS transcripts after TaClo treatment. (c and d) Cropped western blots displaying the levels of the TNF-*α*, IL-6, Cox-2, and iNOS proteins in the nigrostriatal system. ^∗^ *p* < 0.05 and ^∗∗^ *p* < 0.01 compared to the control group. Scale bar 100 *μ*m.

Images of IHC staining showed increased Iba-1 expression after the TaClo treatment, which is a sign of activated microglia. Usually, resting or quiescent microglia exhibit a branched shape with many synapses. Here, numerous resting microglia transformed to activated microglia characterized by a rounded morphology and dark staining which is known as an amoeboid or phagocytic form after the TaClo treatment (Figure 5(a)). Oxidative stress is known to promote the release of many proinflammatory cytokines. Thus, we detected the levels of proinflammatory cytokines in the nigrostriatal system after TaClo treatment. As expected, the expression of the IL-6, TNF-*α*, Cox-2, and iNOS mRNAs was obviously increased (Figure 5(b)). The western blot results also revealed increased levels of IL-6, TNF-*α*, Cox-2, and iNOS protein (Figures 5(c) and 5(d)) as well as increased levels of cleaved caspase-3 in response to TaClo treatment, indicating that cell apoptosis occurred in the nigrostriatal system following treatment.

## 4. Discussion

According to a previous *in vitro* study, TaClo can cause a substantial metabolic disorder in cultured dopaminergic cells [5], as well as DNA damage [15] and apoptosis in human neuroblastoma cells [16]. In the present study, we studied the negative effect of TaClo on dopaminergic neurons *in vivo* by generating a rat model of acute PD through the stereotactic injection of TaClo into the right striatum of Wistar rats. Subsequently, the motor activity of TaClo rats was apparently reduced over time. LC–MS/MS revealed that the motor dysfunction observed in the TaClo-treated rats was accompanied by a decreased level of DA in the striatum. TaClo impaired the function of dopaminergic neurons in the nigrostriatal system by causing demyelination and apoptosis. Further studies investigating the underlying molecular mechanisms discovered that both oxidative stress and the innate inflammatory response were involved in TaClo-induced PD.

**Figure 6:**
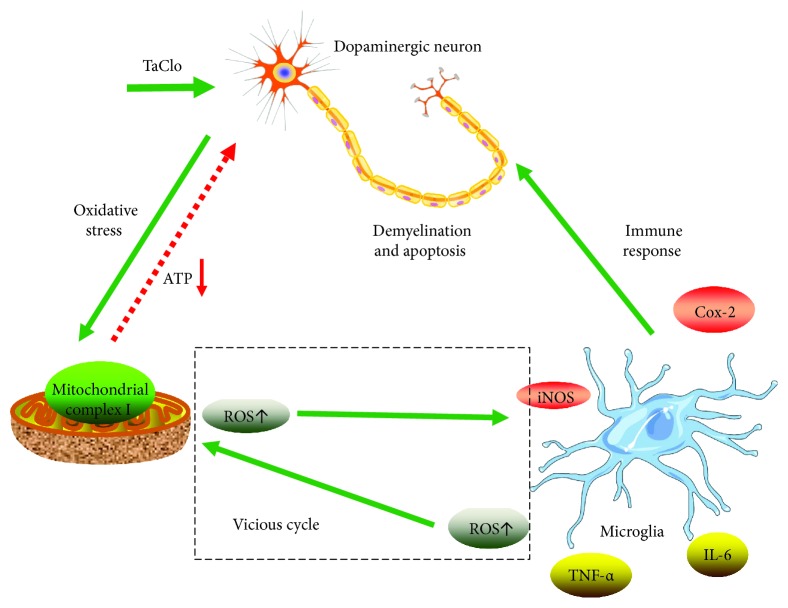
TaClo negatively affected dopaminergic neurons. A schematic diagram showing that TaClo induces the demyelination and apoptosis of dopaminergic neurons by causing mitochondrial dysfunction and triggering the innate immune response in the nigrostriatal system.

The mitochondrion is the primary source of ROS and normally produces a relatively low level of ROS. Oxidative stress increases the ROS level. In the present study, TaClo caused oxidative stress in the mitochondria and disrupted normal ATP production. According to previous research, the underlying mechanism by which TaClo impaired mitochondrial function is due to the highly lipophilic nature of TCE and TaClo. These compounds easily perturb the lipid bilayers of the celluar and mitochondrial membrane [17]. When TaClo accumulates in the CNS, it is taken up by dopaminergic neurons and is transported across the mitochondrial membrane. Then, it will impair mitochondrial complex I activity and block the electron transport chain, which induces mitochondrial dysfunction, including abnormal ATP production, a decrease in the mitochondrial membrane potential, and oxidative stress. A substantial increase in ROS production is involved in triggering the immune and inflammatory responses [13, 18, 19]. Microglia are cells of mesodermal origin in the brain that are activated under pathological conditions including mitochondrial dysfunction and oxidative stress [20]. When overactivated, microglia uncontrollably release large amounts of ROS and proinflammatory cytokines including IL-6, TNF-*α*, Cox-2, and iNOS which could exacerbate the loss of dopaminergic neurons [21]. The enzyme iNOS induces the synthesis of the nitric oxide radical, which aggravates oxidative stress and mitochondrial dysfunction. The mitochondrial oxidative stress caused by TaClo activates the innate immune response by activating microglia. Furthermore, the ROS and proinflammatory cytokines released by microglia can exacerbate the mitochondrial dysfunction. As shown in Figure 6, we propose that a positive feedback mechanism exists between mitochondrial oxidative stress and the innate inflammatory response during the process of TaClo induced PD. This type of loop between oxidative stress and the immune response has been supported by numerous previous articles. Lamb and Goldstein described the interaction between inflammation and oxidative stress as the “oxidative-inflammatory cascade (OIC)” which can lead to many diseases. The OIC is regulated by mediators of the immune and metabolic systems and maintained through a positive feedback loop [22]. According to Voloboueva et al., the mitochondria are both a source of ROS and a critical target of ROS-mediated damage. Mitochondrial ROS production and mitochondrial metabolic disorders are involved in the activation of macrophages called microglia in the CNS [23]. Without any intervention, this vicious cycle can cause serious neural damage since the brain has a lower capacity for cellular regeneration than other organs.

To the best of our knowledge, this study is among the first to identify destruction in the nigrostriatal fibre structure in a neurotoxic rat PD model based on a voxel-based analysis of DTI. ADC values, but not FA values, for the NP were significantly increased after the TaClo treatment. Our findings are consistent with those reported by Hikishima et al. using a marmoset model treated with MPTP [24]. The increased ADC value indicated a significant increase in the radial diffusivity of water molecules within the nigrostriatal fibres, suggesting that demyelination occurs [25–28]. The LFB staining confirmed that demyelination occurred in the TaClo-treated group, but not in the control group consistent with the DTI results. Based on previous research, mitochondrial dysfunction negatively affects the development of oligodendrocytes and even induces apoptosis in oligodendrocytes, which can further damage the myelin sheath [29]. Therefore, the oxidative stress in mitochondria and innate inflammation induced by TaClo are related to the demyelination of dopaminergic neurons in the nigrostriatal system.

Our study also has some limitations. The present TaClo-based PD model did not show the formation of Lewy bodies which is a pathological hallmark of PD. Based on a previous study, the aggregation of *α*-synuclein and the formation of Lewy bodies are chronic processes [30]. Our rat PD model is an acute model. The early stage of PD is not accompanied by Lewy pathology [31]. Therefore, a chronic TaClo-based model is needed in subsequent studies to better simulate the natural course of PD and evaluate the chronic effect of TaClo on the progression and pathological changes in PD.

Regarding human exposure, TaClo-mediated neurotoxic processes extend over years. Kochen et al. reported an association between the onset of PD in three chronic trichloroethylene-exposed individuals and the presence of TaClo in the ng range [32]. Recently, TaClo has been reported to induce epigenetic carcinogenesis [33]. Therefore, TCE exposure is a public risk. Studies of the toxicity of TaClo are very valuable.

## 5. Conclusions

Our study is among the first to investigate the neurotoxic effects of TaClo, such as causing aggressive PD *in vivo*, from the perspective of mitochondrial dysfunction, microglial activation, and the activation of the cascade of inflammatory reactions in the nigrostriatal system. These findings will likely assist with the identification of promising preventative and therapeutic approaches for the treatment of PD.

## Figures and Tables

**Table 1:** 

GADPH	Forward 5′-AACCTGCCAAGTATGATGAC-3′
	Reverse 5′-GCTGTAGCCATATTCATTGT-3′
IL-6	Forward 5′-CTGGAGTTCCGTTTCTACCT-3′
	Reverse 5′–GCCACTCCTTCTGTGACTCT-3′
TNF-*α*	Forward 5′-GCCAATGGCATGGATCTC-3′
	Reverse 5′-TGTCCTTAGGGCAAGGGCT-3′
Cox 2	Forward 5′-CTTCGGAATTCAATCATGAG-3′
	Reverse 5′-TCCAGAACTTCTTTTGAATC-3′
iNOS	Forward 5′-ACATCTGGCAGGATGAGAAG-3′
	Reverse 5′-CTTGTTGTTGAAGGCGTAGC-3′

**Table 2:** Neurotransmitter levels in the rat striatum (mean ± SEM).

	Control	TaClo
Dopamine (ng/g)	11.267 ± 1.079	4.645±1.097^∗∗^
Glutamine (ng/g)	166.533 ± 2.616	169.109 ± 2.992
Glutamate (ng/g)	189.686 ± 1.731	118.114 ± 2.19
5-HT (ng/g)	1.215 ± 0.34	1.399 ± 0.62
5-HIAA (ng/g)	0.148 ± 0.01	0.138 ± 0.028
Acetylcholine (ng/g)	6.37 ± 0.455	6.088 ± 1.61

^∗∗^ *p* < 0.01 compared with the control group.

## Data Availability

The data used to support the findings of this study are available from the corresponding author upon request.

## Supplementary Materials

Supplementary Materials Supplementary File 1: video recording of a TaClo-treated rat's spontaneous rotary motions. (A) On the 28th day, a TaClo-treated rat did a number of spontaneous rotary motions which may be caused by weak sense of balance. Meanwhile, the rat stayed in a corner of the testing box. (B) Video recording of a rat in the control group.

## References

[1] Van der Perren A., Toelen J., Casteels C. (2015). Longitudinal follow-up and characterization of a robust rat model for Parkinson’s disease based on overexpression of alpha-synuclein with adeno-associated viral vectors. *Neurobiology of Aging*.

[2] Sontag T. A., Lange K. W., Heim C., Kolasiewicz W., Tucha O., Sontag K. H. (2009). Alterations of nocturnal activity in rats following subchronic oral administration of the neurotoxin 1-trichloromethyl-1,2,3,4-tetrahydro-*β*-carboline. *Journal of Neural Transmission*.

[3] Bringmann G., Hille A. (1990). Endogenous alkaloids in man, VII: 1-trichloromethyl-1,2,3,4-tetrahydro-*β*-carboline - a potential chloral-derived indol alkaloid in man. *Archiv der Pharmazie*.

[4] Bringmann G., Munchbach M., Feineis D. (2002). Endogenous alkaloids in man. XXXVIII. “chiral” and “achiral” determination of the neurotoxin TaClo (1-trichloromethyl-1,2,3,4-tetrahydro-*β*-carboline) from blood and urine samples by high-performance liquid chromatography–electrospray ionization tandem mass spectrometry. *Journal of Chromatography B, Analytical Technologies in the Biomedical and Life Sciences*.

[5] Bringmann G., Feineis D., Munchbach M. (2006). Toxicity and metabolism of the chloral-derived mammalian alkaloid 1-trichloromethyl-1,2,3,4-tetrahydro-*β*-carboline (TaClo) in PC12 cells. *Zeitschrift für Naturforschung. Section C*.

[6] Janetzky B., God R., Bringmann G., Reichmann H. (1995). 1-Trichloromethyl-1,2,3,4-tetrahydro-beta-carboline, a new inhibitor of complex I. *Journal of Neural Transmission. Supplementum*.

[7] Bringmann G., God R., Feineis D., Janetzky B., Reichmann H. (1995). TaClo as a neurotoxic lead: improved synthesis, stereochemical analysis, and inhibition of the mitochondrial respiratory chain. *Journal of Neural Transmission. Supplementum*.

[8] Huang D., Xu J., Wang J. (2017). Dynamic changes in the nigrostriatal pathway in the MPTP mouse model of Parkinson’s disease. *Parkinson’s Disease*.

[9] Segura-Aguilar J. (2017). Aminochrome as preclinical model for Parkinson’s disease. *Oncotarget*.

[10] Liu M., Shin E. J., Dang D. K. (2018). Trichloroethylene and Parkinson’s disease: risk assessment. *Molecular Neurobiology*.

[11] Waypa G. B., Smith K. A., Schumacker P. T. (2016). O_2_ sensing, mitochondria and ROS signaling: the fog is lifting. *Molecular Aspects of Medicine*.

[12] Liu L., Zhang K., Sandoval H. (2015). Glial lipid droplets and ROS induced by mitochondrial defects promote neurodegeneration. *Cell*.

[13] Amiri S., Haj-Mirzaian A., Momeny M. (2017). Streptozotocin induced oxidative stress, innate immune system responses and behavioral abnormalities in male mice. *Neuroscience*.

[14] Grote C., Clement H. W., Wesemann W. (1995). Biochemical lesions of the nigrostriatal system by TaClo (1-trichloromethyl-1,2,3,4-tetrahydro-beta-carboline) and derivatives. *Journal of Neural Transmission. Supplementum*.

[15] Bringmann G., Munchbach M., Feineis D., Faulhaber K., Ihmels H. (2001). Studies on single-strand scissions to cell-free plasmid DNA by the dopaminergic neurotoxin ‘TaClo’ (1-trichloromethyl-1,2,3,4-tetrahydro-*β*-carboline). *Neuroscience Letters*.

[16] Akundi R. S., Macho A., Munoz E. (2004). 1-Trichloromethyl-1,2,3,4-tetrahydro-*β*-carboline-induced apoptosis in the human neuroblastoma cell line SK-N-SH. *Journal of Neurochemistry*.

[17] Bale A. S., Barone S., Scott C. S., Cooper G. S. (2011). A review of potential neurotoxic mechanisms among three chlorinated organic solvents. *Toxicology and Applied Pharmacology*.

[18] Tseng C. Y., Wang J. S., Chao M. W. (2017). Causation by Diesel Exhaust Particles of Endothelial Dysfunctions in Cytotoxicity, Pro-inflammation, Permeability, and Apoptosis Induced by ROS Generation. *Cardiovascular Toxicology*.

[19] Gurung P., Lukens J. R., Kanneganti T. D. (2015). Mitochondria: diversity in the regulation of the NLRP3 inflammasome. *Trends in Molecular Medicine*.

[20] Marin-Teva J. L., Cuadros M. A., Martin-Oliva D., Navascues J. (2011). Microglia and neuronal cell death. *Neuron Glia Biology*.

[21] Panaro M. A., Cianciulli A. (2012). Current opinions and perspectives on the role of immune system in the pathogenesis of Parkinson’s disease. *Current Pharmaceutical Design*.

[22] Lamb R. E., Goldstein B. J. (2008). Modulating an oxidative-inflammatory cascade: potential new treatment strategy for improving glucose metabolism, insulin resistance, and vascular function. *International Journal of Clinical Practice*.

[23] Voloboueva L. A., Emery J. F., Sun X., Giffard R. G. (2013). Inflammatory response of microglial BV-2 cells includes a glycolytic shift and is modulated by mitochondrial glucose-regulated protein 75/mortalin. *FEBS Letters*.

[24] Hikishima K., Ando K., Yano R. (2015). Parkinson disease: diffusion MR imaging to detect nigrostriatal pathway loss in a marmoset model treated with 1-methyl-4-phenyl-1,2,3,6-tetrahydropyridine. *Radiology*.

[25] Benedetti F., Bollettini I. (2014). Recent findings on the role of white matter pathology in bipolar disorder. *Harvard Review of Psychiatry*.

[26] Lu S. S., Kim S. J., Kim N., Kim H. S., Choi C. G., Lim Y. M. (2015). Histogram analysis of apparent diffusion coefficient maps for differentiating primary CNS lymphomas from tumefactive demyelinating lesions. *American Journal of Roentgenology*.

[27] Song S. K., Sun S. W., Ramsbottom M. J., Chang C., Russell J., Cross A. H. (2002). Dysmyelination revealed through MRI as increased radial (but unchanged axial) diffusion of water. *NeuroImage*.

[28] Branzoli F., Ercan E., Valabregue R. (2016). Differentiating between axonal damage and demyelination in healthy aging by combining diffusion-tensor imaging and diffusion-weighted spectroscopy in the human corpus callosum at 7 T. *Neurobiology of Aging*.

[29] Benedetti F., Poletti S., Hoogenboezem T. A. (2016). Inflammatory cytokines influence measures of white matter integrity in bipolar disorder. *Journal of Affective Disorders*.

[30] Conway K. A., Harper J. D., Lansbury P. T. (1998). Accelerated *in vitro* fibril formation by a mutant *α*-synuclein linked to early-onset Parkinson disease. *Nature Medicine*.

[31] Ferrer I. (2009). Early involvement of the cerebral cortex in Parkinson’s disease: convergence of multiple metabolic defects. *Progress in Neurobiology*.

[32] Kochen W., Kohlmuller D., De Biasi P., Ramsay R. (2003). The endogeneous formation of highly chlorinated tetrahydro-*ß*-carbolines as a possible causative mechanism in idiopathic Parkinson’s disease. *Advances in Experimental Medicine and Biology*.

[33] Sharma R. K., Candelario-Jalil E., Feineis D., Bringmann G., Fiebich B. L., Akundi R. S. (2017). 1-Trichloromethyl-1,2,3,4-tetrahydro-beta-carboline (TaClo) alters cell cycle progression in human neuroblastoma cell lines. *Neurotoxicity Research*.

